# In Vivo, Proteomic, and In Silico Investigation of Sapodilla for Therapeutic Potential in Gastrointestinal Disorders

**DOI:** 10.1155/2019/4921086

**Published:** 2019-12-10

**Authors:** Sameen Fatima Ansari, Arif-ullah Khan, Neelum Gul Qazi, Fawad Ali Shah, Komal Naeem

**Affiliations:** Riphah Institute of Pharmaceutical Sciences, Riphah International University, Islamabad, Pakistan

## Abstract

This study aims to delineate the effects of *Manilkara zapota* Linn. (Sapodilla) fruit chloroform (Mz.CHCl_3_) and aqueous (Mz.Aq) extracts tested through different techniques. Antidiarrheal activity and intestinal fluid accumulation were examined by using castor oil-induced diarrhea and castor oil fluid accumulation models. Isolated rabbit jejunum tissues were employed for in vitro experiments. Antimotility and antiulcer were performed through charcoal meal transient time and ethanol-induced ulcer assay, molecular studies were conducted through proteomic analysis, and virtual screening was performed by using a discovery studio visualizer (DSV). Mz.CHCl_3_ and Mz.Aq extracts attributed dose-dependent (50–300 mg/kg) protection (20–100%) against castor oil-induced diarrhea and dose-dependently (50–300 mg/kg) inhibited intestinal fluid secretions in mice. Mz.CHCl_3_ and Mz.Aq extracts produce relaxation of spontaneous and K^+^ (80 Mm) induced contractions in isolated tissue preparations and decreased the distance moved by charcoal in the gastrointestinal transit model in rats. It showed gastroprotective effect in ulcerative stomach of rats and decreased levels of IL-18 quantified by proteomic analysis. Histopathological results showed ethanol-induced significant gastric injury, leading to cloudy swelling, hydropic degeneration, apoptosis, and focal necrosis in all gastric zones using hematoxylin and eosin (H&E) staining. Moreover, ethanol increased the activation and the expression of tumor necrotic factor (TNF-*α*), cyclooxygenase (COX-2), and nuclear factor kappa-light-chain-enhancer of activated B cells (p-NF*κ*B). In silico results were comparative to in vitro results evaluated through virtual screening. Moreover, ethanol increased the activation and expression of tumor necrotic factor, cyclooxygenase, and nuclear factor kappa-light-chain-enhancer of activated B cells. This study exhibits the gastroprotective effect of *Manilkara zapota* extracts in the peritoneal cavity using a proteomic and in silico approach which reveals different energy values against target proteins, which mediate the gastrointestinal functions.

## 1. Introduction

Gastrointestinal disorders are highly prevalent among the people of Asia. Health physicians claimed that it is an origin for the occurrence of several other comorbidities. Till now, there is no significant drug that has been discovered for gastrointestinal disorders, which completely eradicates the disease [1]. Moreover, herbal therapies have shown excellent economical and long-lasting potential to treat gastric system disorders [2].

In silico and in vivo evaluation of fruit extracts helps to screen out novel bioactive molecules, and their further processing leads to the development of innovative therapies. Most significant and desirable therapeutic effects can be achieved with the purified form of isolated bioactive constituents that can be formulated into suitable dosage form along with the dosage regimen. Many chronic disorders have been treated by herbal remedies, particularly by consuming fruits as functional foods as well as their active constituents. Researchers have investigated the role of crude fruit extracts, which have the potential to combat gastrointestinal disorders.

*Manilkara zapota* Linn. commonly known as Sapodilla or Naseberry is a terrestrial photosynthetic epiphytic plant with a Mediterranean distribution and about 8–15 m in height, and it belons to the family Sapotaceae; tillage is found easily throughout the regions of Asia, though *Manilkara zapota* Linn. originates in Mexico and Central America [3]. Fruits are edible and sweet with rich fine flavor and seeds are aperients, diuretic tonic, and febrifuge. Barks and leaves are used as tonic to treat cough, cold, diarrhea, dysentery, and paludism. Various constituents were isolated from *Manilkara zapota* L. which are methyl chlorogenate, caffeic acid, dihydromyricetin, quercitrin, 4‐O‐galloylchlorogenic acid, myricetin-3-O-*α*-L-rhamnoside, (+)‐catechin, apigenin-7-O-*α*-L-rhamnoside, (−)‐epicatechin, (+)‐gallocatechin, methyl 4‐O‐galloylchlorogenate, and gallic acid [4, 5] along with some novel triterpenes that have been identified as 4-caffeoylquinic acid (cryptochlorogenic acid), lupeol-3-acetate, methyl 4-O-galloylchlorogenate, and *β*-amyrin-3-(3′-dimethyl) butyrate [6]. Sapotin and escitalopram extracted from the fruit pulp are used as antipyretics and antidepressants, respectively [7].

**Figure 1:**
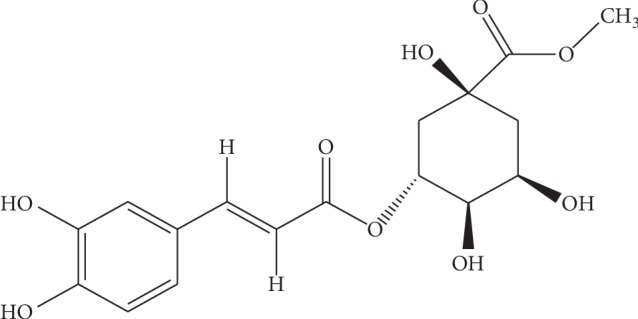
2D chemical structure of *Manilkara zapota* constituent (methyl chlorogenate) drawn through GaussView 5.0 Software and saved in PDB format.

This research aimed to delineate *Manilkara zapota* fruit extracts for antidiarrheal effects, antisecretory effects, isolated tissue preparation, gastrointestinal transient time, and antiulcer effects. On the basis of ethnomedicinal uses, we assumed that extracts modulate the expression of proteins in the stomach and thus potentially promote the molecular and organ/tissue damage associated with diseases [5]. Ulcer is an inflammatory disorder, so we hypothesize here whether *Manilkara zapota* extract could cure gastric ulcer by inhibiting NF*κ*B-dependent proinflammatory cytokines. The present study aims to evaluate whether *Manilkara zapota* effects on inflammation and oxidative stress could eventually account for gastric cell protection. Moreover, in silico approach helps us to understand the biochemical mechanisms and unknot the complex signaling network, which controls cellular function including apoptosis and inflammation [8]. The chemical structure of *Manilkara zapota* constituent is shown in Figure 1.

## 2. Materials and Methods

### 2.1. Materials

*Manilkara zapota* fruits (4 kg) were purchased from the local market in March 2018 and verified by a taxonomist Dr. Mushtaq Ahmad, at the Department of Plant Sciences, Quaid-a-Azam University, Islamabad. Voucher specimen no. ISL-B-23 was collected after submitting the specimen sample of species to the herbarium. Plants were dried, crushed, and extracted with chloroform (4 × 500 mL). The whole extracts were filtered through a standard procedure using Whatman No. 1 filter paper and then evaporated through a rotary shaker (Tokyo Rikakikai Type A 1000S) under reduced pressure at temperature not exceeding 55°C to yield 30 g dry extract. The leftover residue was suspended in 4 L of methanol-water (20 : 80) and successively extracted with the aforementioned technique. The solvent was evaporated under reduced pressure to yield 110 g of extract [5].

### 2.2. Chemicals

Atropine sulphate, acetylcholine, activated charcoal, ethanol, loperamide, methanol, omeprazole, papaverine, potassium chloride, and verapamil hydrochloride (Sigma Chemicals Co, St Louis, MO, USA) were used. Castor oil was obtained from KCL Pharma, Karachi, Pakistan.

### 2.3. Experimental Animals and Housing Conditions

Experiments were performed in compilation with the rules of Research and Ethics Committee of RIPS (Ref. no. REC/RIPS/2018/021) along with the guidelines of “Principles of Laboratory Animal care.” Balb/C mice (20–25 g), rabbits (1.0–2.0 kg), and Sprague-Dawley rats (190–240 g) of either gender were used in the experimentation procedure and were kept in animal house of the Riphah Institute of Pharmaceutical Sciences (RIPS), Islamabad, provided with controlled environment (20–25°C).

### 2.4. Phytochemical Analysis

Preliminary phytochemical analysis was carried out for the detection of major secondary metabolites such as saponins, glycosides, anthraquinones, alkaloids, steroids, proteins, flavonoids, and tannins according to the standard procedure with slight modifications [9].

### 2.5. Castor Oil-Induced Diarrhea

The protocol was previously described by Sisay et al. [10]. Balb/C mice were randomly allocated to five groups for Mz.CHCl_3_ and Mz.Aq, respectively. Animals were fasted for 24 hours (08:00–08:00) before experimentation. Each floor of cage was lined with blotting sheets in which animals were placed. Negative control group received normal saline (10 mL/kg) while positive control group received loperamide hydrochloride (2 mg/kg), and the remaining groups were given 50, 100, and 300 mg/kg of the extracts orally, respectively. After 1 hour of treatment, all mice received castor oil (10 mL/kg p.o.). Animals were individually observed for a period of 4 hours to analyze the onset of diarrhea and presence and absence of diarrheal dropping. The percentage of diarrheal protection was evaluated according to Rehman et al. [11] by using the chi-square (*Χ*^2^) test.

### 2.6. Assessment of Intestinal Fluid Accumulation

Intestinal fluid accumulation was determined using the method described by Sisay et al. [10]. To study the intestinal fluid accumulation, enteropooling assay was used. Mice fasted overnight for 24 hours (08:00–08:00) were taken and placed in five assigned cages (*n* = 5). Groups I and II were given normal saline (10 mL/kg) and castor oil (10 mL/kg, p.o.), respectively. Mz.CHCl_3_ doses of 50, 100, and 300 mg/kg intraperitoneally were administered to Groups III, IV, and V, respectively. Standard drug atropine at dose 10 mg/kg was given to II group, 1 hour prior induction with castor oil (10 mL/kg, p.o.). The same procedure was repeated for the Mz.Aq extract. Mice were sacrificed after 30 min, and the intestine was removed and weighed. The results were articulated as (*P*_i_/*P*_m_) × 1000, where *P*_i_ is the weight (g) of the intestine and *P*_m_ is the weight of the animal, by using GraphPad Prism and analysis of variance one-way ANOVA followed by Tukey's post hoc test.

### 2.7. Isolated Tissue Preparation

Before experimentation, the rabbit was fasted for 24 hours (08:00–08:00) but had a free access to water. After cervical dislocation, jejunal portion of 2 cm was isolated and washed with Tyrode's solution and suspended in tissue bath containing Tyrode's solution to equilibrate for 30 minutes with environment along with proper supply of oxygen (95% O_2_) and 5% CO_2_ (carbogen) under control temperature conditions before drug administration. Each preparation was stabilized with concentration of ACh (0.3 *μ*M), until constant responses were recorded via a force displacement transducer (model FT-03) coupled with a bridge amplifier and a power Lab 4/25 data acquisition system connected to computer running Lab-Chart 6 software (AD Instrument, Sydney, Australia). Graded doses of Mz.CHCl_3_ and Mz.Aq extracts were tested for % change in contractions of jejunum [12]. The graphs were analyzed by using the nonlinear regression curve followed by sigmoidal dose-response (variable slope) by using GraphPad Prism.

### 2.8. Charcoal Meal Transit Time

The inhibitory activity of the propulsion of a charcoal meal was estimated as described by Shang et al. [13]. Rats were fasted for 24 hours but had a free access to water. Test groups received the doses 50, 100, and 300 mg/kg of Mz.CHCl_3_ and Mz.Aq according to body weight, where they as positive control group received atropine sulphate (0.1 mg/kg, i.p.), while the negative control group received normal saline (10 mL/kg, p.o.). After one hour, animals receive charcoal meal (1 mL/kg). 30 minutes after all treatments, all the animals were sacrificed. The small intestine was excised, and the distance travelled by charcoal meal (5% activated charcoal suspension in distilled water) through the organ was expressed as a percentage of the length of the small intestine according to the following expression. The data were analyzed by using one-way ANOVA followed by Tukey's post hoc test:

(1) $$\begin{array}{c} \text{peristaltic index }\left(\text{PI}\right)\%=\frac{\text{distance moved by charcoal meal}}{\text{total length of the intestine}}\times 100. \end{array}$$

For further evaluation of % inhibition, peristaltic index is used:

(2) $$\begin{array}{c} \%\text{ inhibition }=\frac{\left(\text{PIC}-\text{PIT}\right)}{\text{PIC}}\times \;100, \end{array}$$

where PIC is the peristaltic index of control and PIT is the peristaltic index of the test group.

### 2.9. Ethanol-Induced Ulcer Assay

Rats were randomly assigned into groups weighing 190–240 g of either sex and kept for fasting period of 24 hours (09:00–09:00). Group 1 served as a negative control received normal saline 10 mL/kg body weight, and group 2 received 20 mg/kg (p.o.) omeprazole as standard drug. Groups 3, 4, and 5 received 50, 100, and 300 mg/kg (p.o.) of Mz.CHCl_3_ and Mz.Aq extracts, respectively. All the animals were treated with 1 mL/100 g of ethanol (p.o.) after 1 hour of treatment. Animals were sacrificed through cervical dislocation and stomachs were removed and washed with normal saline, and lesion index was estimated by measuring each lesion in mm along its greater curvature. Surface area of each lesion was measured and scoring was done. For each stomach lesion, ulcer index was taken as mean ulcer score (US) (such as 0: no ulcer, 1: US ≤ 0.5 mm^2^, 2: 0.5 < US ≤ 2.5 mm^2^, 3: 2.5 mm^2^ < US ≤ 5 mm^2^, 4: 5 mm^2^ < US ≤ 10 mm^2^, 5: 10 mm^2^ < US ≤ 15 mm^2^, 6: 15 mm^2^ < US ≤ 20 mm^2^, 7: 20 mm^2^ < US ≤ 25 mm^2^, 8: 25 mm^2^ < US ≤ 30 mm^2^, 9: 30 mm^2^ < US ≤ 35 mm^2^; and 10: US > 35 mm^2^) [14]. For each stomach injury, the sum of the lengths (mm) of all scores was utilized as the ulcer index (UI). The gastroprotective assessment was displayed as an inhibition percentage (*I*%) calculated by the following formula:

(3) $$\begin{array}{c} \%\text{inhibition}=\;\frac{\left(U_{\text{Sc}}-U_{\text{St}}\right)}{U_{\text{Sc}}\;}\times \;100, \end{array}$$

where *U*_Sc_ is the ulcer surface area of control and *U*_St_ is the ulcer surface area of the test drug group.

The data were analyzed by using one-way ANOVA followed by Tukey's post hoc test. The gastric tissues were stored in a biofreezer (−80°C) for further proteomic analysis.

### 2.10. Enzyme-Linked Immunosorbent Assay (ELISA)

IL-18 expression was measured using Rat IL-18 ELISA kit (E-EL-R0567) according to the manufacturer's instructions (Elabscience). The tissues were homogenized at 15000 using Silent Crusher M (Heidolph), and the supernatant was collected after centrifugation (at 1350 ×g for 1 h). The concentration of IL-18 was determined by using an ELISA microplate reader [15]. Values are expressed as pictograms of cytokines per milliliter (pg/mL) and evaluated by using two-way ANOVA followed by Tukey's post hoc test.

### 2.11. Hematoxylin and Eosin (H&E) Staining and Immunohistochemical Analysis

Tissue sections on coated slides were deparaffinized with absolute xylene (100%) and rehydrated with ethyl alcohol (from 100% (absolute) to 70%). The slides were rinsed with distilled water and immersed in hematoxylin for 10 min. The slides were then kept under running water in glass jar for 10 minutes and treated with 1% HCl and 1% ammonia water as previously reported by Gim et al. [16]. The slides were added to eosin solution for 5–10 min. After due time, the slides were rinsed in water and air-dried for some time. The dried slides were dehydrated in graded ethyl alcohol (70%, 95%, and 100%). The slides were cleared with xylene and were mounted with glass cover slip. The slides were pictured with light microscope (Olympus, Japan) and analyzed by ImageJ, a computer-based program. The number of images per slide was five per group, while focusing specifically on gastric cell size and shape, inflammatory infiltrated cells, and vacuolation described by Shah et al. [8]. The TIF images were optimized to the same threshold intensity for all groups and examined in GraphPad Prism.

Immunohistochemical staining was performed as described previously with little modifications done by Gim et al. [16]. After deparaffinization, slides were processed for antigen retrieval step (enzymatic method) and then washed with PBS. The endogenous peroxidase was quenched by applying 3% hydrogen peroxide (H_2_O_2_) in methanol for 10 min. The slides were incubated with 5% normal goat serum containing 0.1% Triton X-100. After blocking, the slides were incubated overnight with mouse anti-TNF-*α*, p-NF*κ*B, and mouse anti-COX-2 antibodies (dilution 1 : 100, Santa Cruz Biotechnology). The following morning, after washing with 0.1 M PBS, slides were incubated in biotinylated secondary antibody (dilution 1 : 50) according to the origin of the primary antibody and serum used. Following secondary antibody treatment, slides were incubated with ABC Elite kit (Santa Cruz Biotechnology) for 1 hour in a humidified chamber. The slides were washed with 0.1 M PBS, stained in DAB solution, washed with distilled water, dehydrated in a graded ethanol series, fixed in xylene, and cover-slipped in mounting medium. Immunohistochemical TIF images (five images per slide) were captured with a light microscope. ImageJ software was used to quantitatively determine hyperactivated p-NF*κ*B, COX-2, and TNF-*α*, by optimizing the background of images according to the threshold intensity and analyzing p-NF*κ*B, COX-2, and TNF-*α* positive cells at the same threshold intensity for all groups. The intensity is expressed as the relative integrated density of the samples relative to the saline by using one-way ANOVA followed by Tukey's post hoc test.

### 2.12. Acute Toxicity

This study delineates lethal versus nonlethal dose of extracts in the animal model by using acute toxicity model. Mice were divided into three groups of five mice each. The test was performed using increasing doses (3 and 5 g) of the Mz extract given in 10 mL/kg volume. Saline (10 mL/kg, p.o., negative control) was administered to one group. Animals were observed for change in behavior for about 4 hours, and mortality rate was studied for 24 hours after drug administration described by Ogwal-Okeng et al. [17].

### 2.13. Computational Studies

Through DSV, 3-dimensional structures of reference drugs were prepared (2016) by adding polar hydrogen atoms (H-atoms) and converted into PDB file and downloaded from PubChem database. Standard drugs for docking included phenylephrine, pirenzepine, atropine, domperidone, calmidazolium, verapamil, omeprazole, ranitidine, loperamide, and papaverine for receptors. Targets involved in gut physiology were selected and obtained from the website of RCSB protein data bank. Selected targets included adrenergic *α*_1_ receptor (PDB ID: P35348), calmodulin (PDB ID: 1CTR), dopaminergic D_2_ (PDB ID: 6CM4), histaminergic H_2_ (PDB ID: P25021), H^+^/K^+^ ATPase (PDB ID: 4UX2), muscarinic M_1_ (PDB ID: 5CXV), muscarinic M_3_ (PDB ID: 2CSA), mu-opioid (PDB ID: 6DDE), phosphodiesterase enzyme (PDB ID: 6DDE), and voltage gated L-type calcium channel (PDB ID: 1T3S). Structures were purified by removing water molecules and ligands in DSV and were saved in PDB format till further procedure. PyRx is used to perform molecular docking, and the results were analyzed through the best pose by using DSV and energy values (kcal/mol) achieved through PyRx in sdf and csv formats. Only one pose having the lowest value of energy (kcal/mol) was selected for postdock analysis through DSV after the evaluation of top ten conformations. 2D image was assessed to check the ligands and amino-acid residue interactions with the receptor including alanine (ALA), asparagine (ASN), aspartic acid (ASP), arginine (ARG), lysine (LYS), threonine (THR), glycine (GLY), glutamine (GLN), cysteine (CYS), methionine (MET), glutamic acid (GLU), histidine (HIS), phenylalanine (PHE), isoleucine (ILE), proline (PRO), tyrosine (TYR), serine (SER), threonine (THR), tryptophan (TRP), and valine (VAL). Docking signifies the binding of the receptor with the ligand in the physiological mechanism and hence provides a valuable marker for drug binding inside the body.

### 2.14. Statistical Analysis

Data were expressed as mean ± SEM (*n* = 5) and median effective concentrations (EC_50_) having 95% confidence intervals. Statistical analysis of the results was analyzed using one-way ANOVA followed by Tukey's post hoc test. Chi-square test was used in the case of the antidiarrheal data, where *p* < 0.05 was regarded as significant. Nonlinear regression using GraphPad program (GraphPAD, San Diego, CA, USA) was used to analyze the concentration-response curves.

## 3. Results

### 3.1. Phytochemical Profile

Qualitative phytochemical analysis of Mz.CHCl_3_ and Mz.Aq showed the presence of flavonoids, alkaloids, phenols, saponins, triterpenes, lignin, unsaturated sterols, and carbohydrates validated through chemical assays.

### 3.2. Effect of Mz.CHCl_3_ and Mz.Aq on Castor Oil-Induced Diarrhea

Mz.CHCl_3_ and Mz.Aq exhibited dose-dependent (50–300 mg/kg) protective effect against castor oil-induced diarrhea in mice, and the negative control group (saline treated) did not show any protection against castor oil-induced diarrhea. *Manilkara zapota* extract showed marked response of 60%, 80%, and 100 in Mz.CHCl_3_ and 20%, 40%, and 60% in Mz.Aq at doses of 50, 100, and 300 mg/kg, respectively (^*∗*^*p* < 0.05 versus saline group). Loperamide hydrochloride (2 mg/kg), a well-known antidiarrheal medicine, showed 100% protection from diarrhea (^*∗∗*^*p* < 0.01 versus saline group) in the positive control group (Table 1).

### 3.3. Effect of Mz.CHCl_3_ and Mz.Aq on Enteropooling Assay

**Figure 2:**
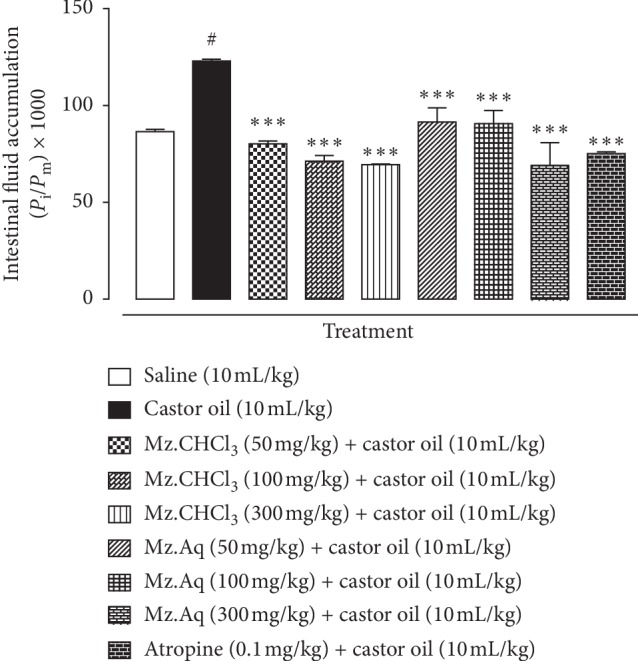
Inhibitory effect of *Manilkara zapota* extracts: chloroform (Mz.CHCl_3_), aqueous (Mz.Aq), and atropine on castor oil-induced fluid accumulation in mice. Results are expressed as mean ± SEM, *n* = 5. Antisecretory effect is expressed as *P*_i_/*P*_m_ × 1000 g, where *P*_i_ is the weight of the small intestine and *P*_m_ is the weight of mouse; ^#^*p* < 0.001 versus saline group, ^*∗∗∗*^*p* < 0.001 versus castor oil group, one-way analysis of variance with Tukey's post hoc test.

When tested against castor oil-induced enteropooling assay in mice, Mz.CHCl_3_ exhibited a dose-dependent (50–300 mg/kg) antisecretory effect. Intestinal fluid accumulation in the saline treated group was 86.5 ± 0.93 (mean ± SEM, *n* = 5), whereas in the castor oil-treated group it was 122.96 ± 0.93 (^*∗∗∗*^*p* < 0.001 versus saline group). Mz.CHCl_3_ at the doses of 50, 100, and 300 mg/kg reduced the castor oil-induced fluid accumulation to 109.29 ± 2.62 (^*∗∗∗*^*p* < 0.001 versus castor oil group), 94.78 ± 3.09 (^*∗∗∗*^*p* < 0.001 versus castor oil group), and 77.68 ± 3.33 (^*∗∗∗*^*p* < 0.001 versus castor oil group), respectively, while the effect of Mz.Aq at the doses of 50, 100, and 300 mg/kg reduced the castor oil-induced fluid accumulation to 104.28 ± 3.08 (^*∗∗∗*^*p* < 0.001 versus castor oil group), 95.58 ± 3.11 (^*∗∗∗*^*p* < 0.001 versus castor oil group), and 89.77 ± 1.62 (^*∗∗∗*^*p* < 0.001 versus castor oil group), respectively. Atropine at the dose of 10 mg/kg decreased the intestinal fluid accumulation to 75.11 ± 0.42 (^*∗∗∗*^*p* < 0.001 versus castor oil group), as shown in Figure 2.

### 3.4. Effect of Mz.CHCl_3_ and Mz.Aq on Spontaneous and K^+^-Induced Contractions

**Figure 3:**
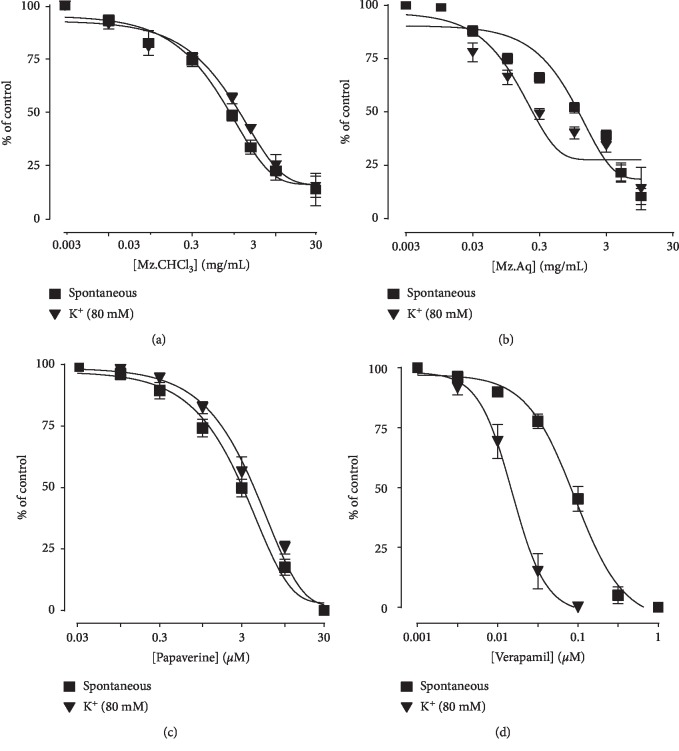
Concentration-dependent inhibitory effect on spontaneous and K^+^ (80 mM) induced contractions of *Manilkara zapota* extracts: (a) chloroform (Mz.CHCl_3_), (b) aqueous (Mz.Aq), (c) papaverine, and (d) verapamil in isolated tissue preparations. Results are expressed as mean ± SEM (*n* = 3–5).

Figure 3 shows the inhibitory effect of the plant extract, papaverine, and verapamil against spontaneous and K^+^ (80 mM) induced contractions. Mz.CHCl_3_ was found to be equally effective against spontaneous and K^+^ (80 mM) induced contractions with EC_50_ values of 2.170 mg/mL (1.676–2.810, *n* = 4) and 5.644 mg/mL (1.697–18.77, *n* = 4), respectively, as shown in Figure 3(a). Similarly, Mz.Aq showed spontaneous and K^+^ (80 mM) induced contractions with EC_50_ values of 0.7412 mg/mL (0.1139–4.822, *n* = 4) and 0.1669 mg/mL (0.03844–0.7251, *n* = 4), respectively, as shown in Figure 3(b). Papaverine also showed a similar pattern of nonspecific inhibitory response (Figure 3(c)) with respective EC_50_ values of 0.4 *μ*M (0.2–0.8, *n* = 4) in spontaneous and 0.6 (0.3–1.3, *n* = 4) in high K^+^-induced contractions, whereas verapamil was found more potent against K^+^ (80 mM) induced contractions with EC_50_ value of 0.04 *μ*M (0.03–0.06, *n* = 4), as compared to spontaneous contractions (0.12 *μ*M (0.10–0.20, *n* = 3)), as shown in Figure 3(d).

### 3.5. Effect of Mz.CHCl_3_ and Mz.Aq on Charcoal Meal Transit Time

Mz.CHCl_3_ delays the charcoal meal to travel through the small intestine in a dose-dependent manner. The distance travelled by the saline group was 92.6%. Mz.CHCl_3_ at 50, 100, and 300 mg/kg dose showed % inhibition in charcoal meal transit by 9.74, 19.92, and 27.03%, respectively, whereas Mz.Aq showed % inhibition of 12.48%, 20.89%, and 25.91% at doses of 50, 100, and 300 mg/kg, respectively (^*∗*^*p* < 0.05, ^*∗∗*^*p* < 0.01, and ^*∗∗∗*^*p* < 0.001 versus saline group). Atropine (0.1 mg/kg, i.p.) shows inhibitory effect of 81.40% (Table 2).

### 3.6. Effect of Mz.CHCl_3_ and Mz.Aq on Ethanol-HCl-Induced Ulcer

**Figure 4:**
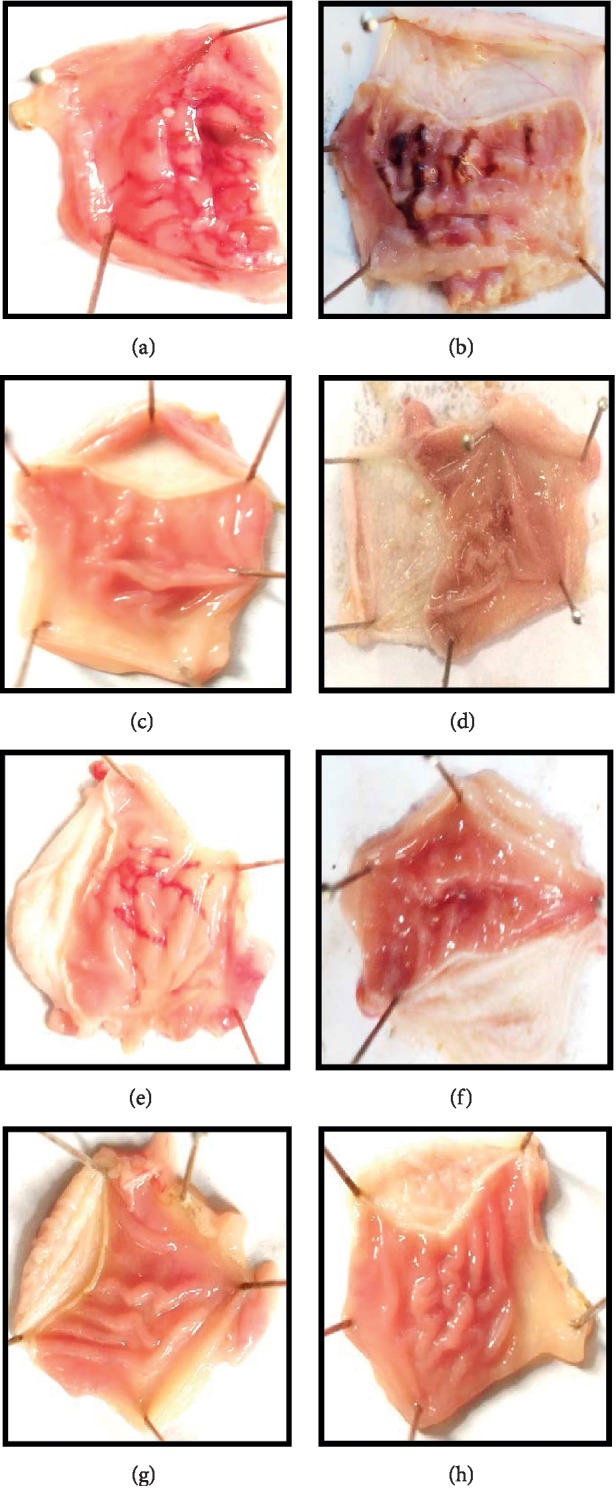
Gross-appearance of gastric mucosa in rats: (a) pretreated with saline, 10 mL/kg, severe injuries are seen, as ethanol (1 mL/100 g) produced excessive hemorrhagic necrosis of gastric mucosa pretreated with *Manilkara zapota* extracts: (b–d) chloroform (Mz.CHCl_3_), (e–g) aqueous (Mz.Aq) at doses of 50, 100, and 300 mg/kg, respectively, and (h) pretreated with omeprazole (20 mg/kg). The injuries were reduced with the increase of Mz.CHCl_3_ and Mz.Aq doses and omeprazole compared with ulcer control. At 300 mg/kg, Mz.CHCl_3_ and Mz.Aq showed most efficacious gastroprotective action.

Mz.CHCl_3_ in dose-dependent manner (50–300 mg/kg) exhibited an antiulcer effect. Mz.CHCl_3_ at 50, 100, and 300 mg/kg caused 20, 58, and 76% (^*∗∗∗*^*p* < 0.001 versus ethanol group) inhibition, respectively. Omeprazole (20 mg/kg) exhibited 86% inhibitory effect. Mz.Aq showed antiulcer effect in healing wound at doses of 50, 100, and 300 mg/kg and % inhibition was 38, 56, and 76%, respectively (Table 3). Macroscopic observation showed the gastric mucosa of rats (Figure 4).

### 3.7. Effect of Mz.CHCl_3_ and Mz.Aq on IL-18 Expression in the Stomach of Ethanol-Induced Ulcer Models

**Figure 5:**
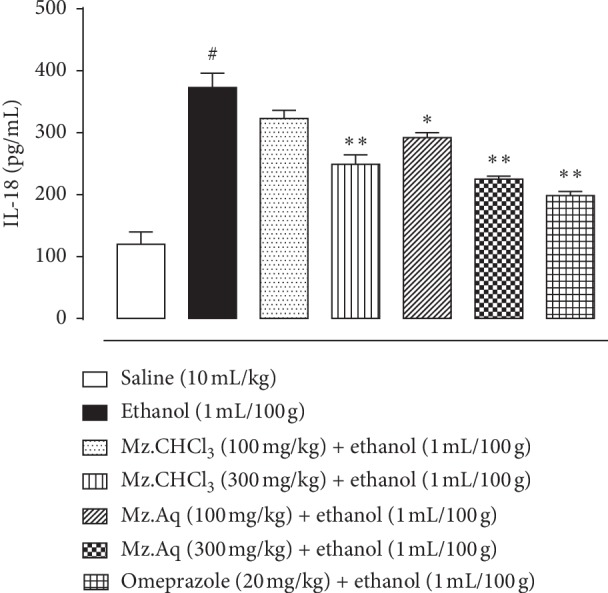
Effect of *Manilkara zapota* extracts: chloroform (Mz.CHCl_3_) and aqueous (Mz.Aq) in comparison with saline, ethanol, and omeprazole groups on IL-18 in ethanol-induced ulcer model. IL-18 levels were measured by ELISA assay. ^*∗*^*p* < 0.05, ^*∗∗*^*p* < 0.01, and ^#^*p* < 0.001. Analyzed by one-way ANOVA followed by Tukey's post hoc test.

Figure 5 illustrates the effect of *Manilkara zapota* chloroform (Mz.CHCl_3_) and aqueous extract (Mz.Aq) on IL-18 in gastric tissues. One-way analysis of variance (ANOVA) followed by Tukey's post hoc test revealed that *Manilkara zapota* extracts significantly reduced the IL-18 expression in treated groups as compared to the negative control group (^*∗*^*p* < 0.05 and ^*∗∗*^*p* < 0.01 versus ethanol group) (^#^*p* < 0.001 versus saline). The levels are significantly decreased in treated versus disease group. In Mz.CHCl_3_ at doses of 100 and 300 mg/kg, the level of inflammatory mediator release from the tissue was relatively less at higher doses, in contrast to the response shown by Mz.Aq at doses of 100 and 300 mg/kg. In comparison, Mz.Aq has more significant results with respect to omeprazole.

### 3.8. Histopathology and Immunohistochemistry (IHC) Analysis

**Figure 6:**
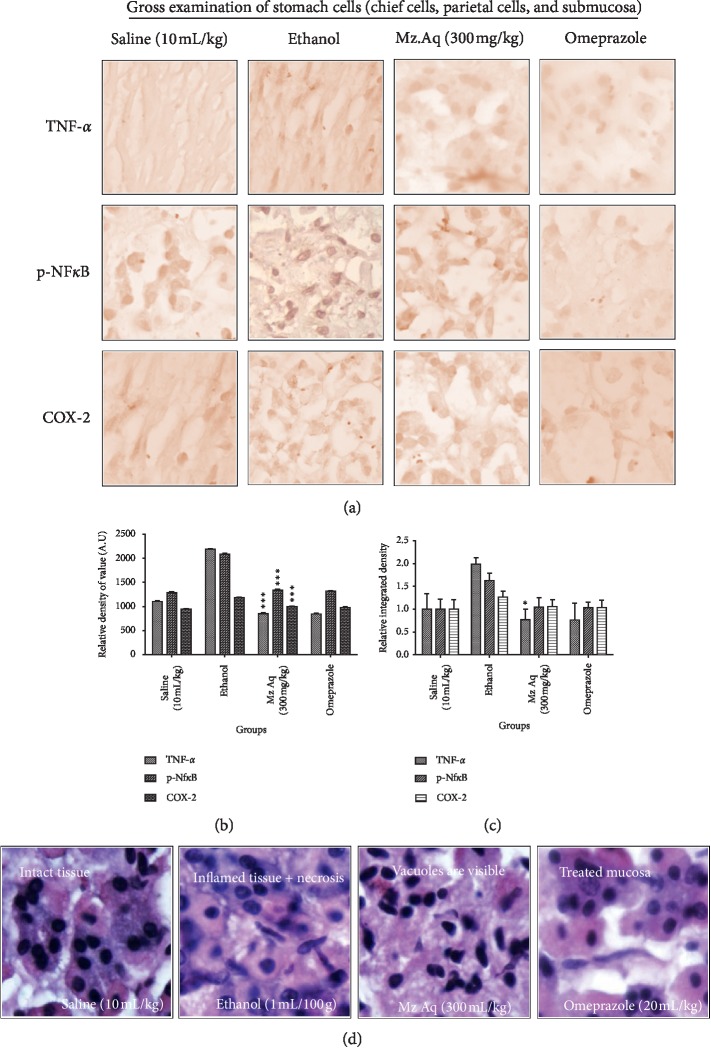
Effects of Mz.Aq extract on ethanol-mediated stomach histopathologic changes. Stomach tissues (*n* = 5) from each experimental group were processed for histological evaluation at 1 hour after ethanol challenge. (a) Representative histological changes of apoptotic markers (TNF-*α*, P-NF*κ*B, and COX-2) in stomach, scale bar = 20 *μ*m, magnification 40x: control group, ethanol group, Mz.Aq extract group, and omeprazole group. (b, c) Severity scores of stomach injury in different groups (*n* = 5) calculated via relative density of value (AU) and relative integrated density. Data were analyzed by two-way ANOVA followed by Tukey's post hoc test using GraphPad Prism software. Mean ± SEM. ^*∗∗∗*^*p* < 0.001 versus ethanol group; ^*∗*^*p* < 0.01 versus ethanol group. (d) Histopathological examination of saline group, ethanol group, test group, and omeprazole group.

Figure 6 shows IHC and H&E staining in order to distinguish necrotic cells from intact ones. Ethanol produces robust cellular changes in the highly prone areas of gastric cells, while treatment with Mz.Aq extracts attenuates this damage. A substantial difference was observed in the control group relative to the ethanol-administered group. The damaged area exhibits abnormal morphological features including alteration in mucosal cell size and shape, alteration in color staining (cytoplasmic eosinophilia/pyknosis and basophilic nature of nucleus), and vacuolation. No noticeable alterations were noticed in the histological preparations of control animals. Significantly more intact cells were there in the Mz.Aq extract treated groups compared to the ethanol-operated group (^*∗*^*p* < 0.05, ^*∗∗*^*p* < 0.01, and ^*∗∗∗*^*p* < 0.001). Severity scores of stomach injury in different groups (*n* = 5) are calculated via relative density of value (AU) and relative integrated density. Data are mean ± SEM. ^*∗∗∗*^*p* < 0.001 versus ethanol group; ^*∗*^*p* < 0.05 versus ethanol group.

### 3.9. Acute Toxicity

The extracts did not cause any significant behavioral and pathological changes and did not show any mortality up to the dose of 5 g/kg.

### 3.10. Docking Evaluation

**Figure 7:**
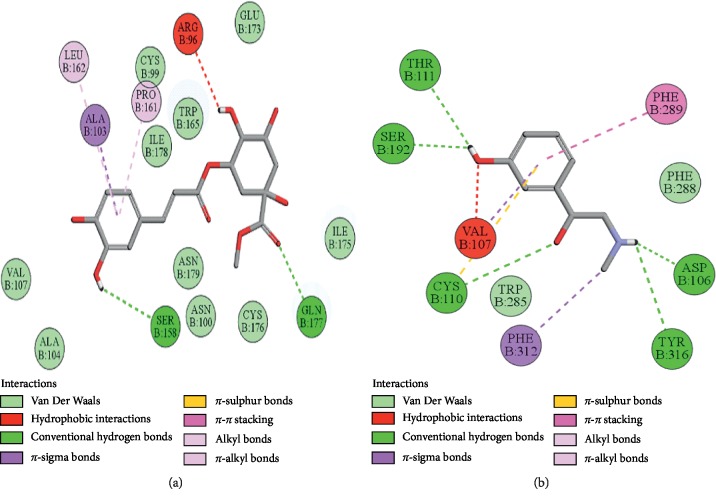
(a, b) The interactions of methyl chlorogenate and phenylephrine against the target adrenergic *α*_1_ receptor, respectively, evaluated through Biovia Discovery Studio 2016.

**Figure 8:**
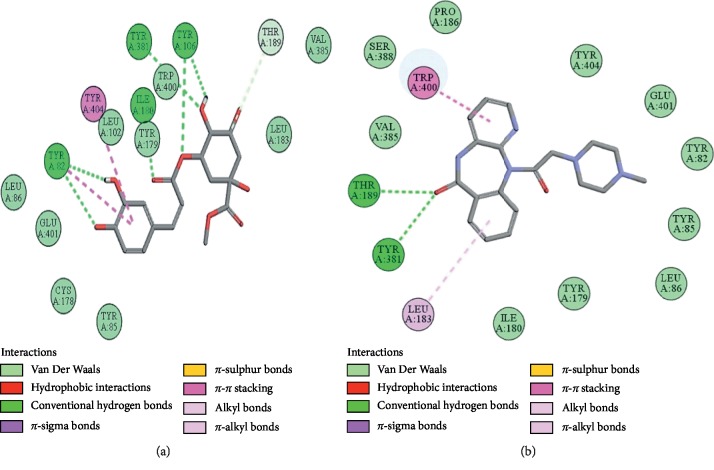
(a, b) The interactions of methyl chlorogenate and pirenzepine against the target muscarinic M_1_ receptor, respectively, evaluated through Biovia Discovery Studio 2016.

**Figure 9:**
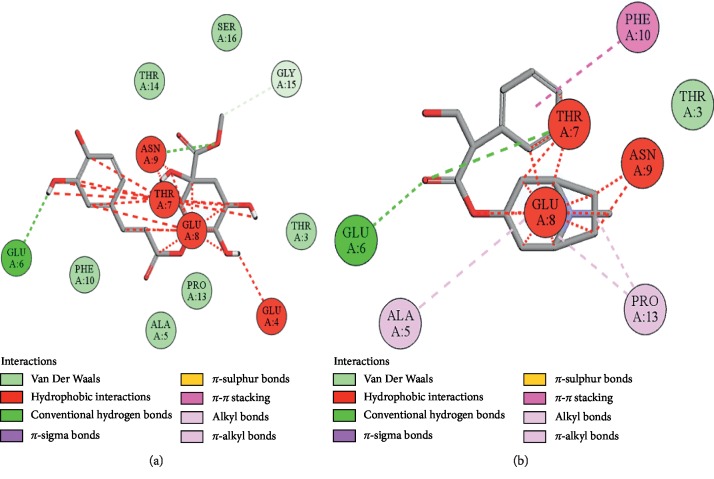
(a, b) The interactions of methyl chlorogenate and atropine against the target muscarinic M_3_ receptor, respectively, evaluated through Biovia Discovery Studio 2016.

**Figure 10:**
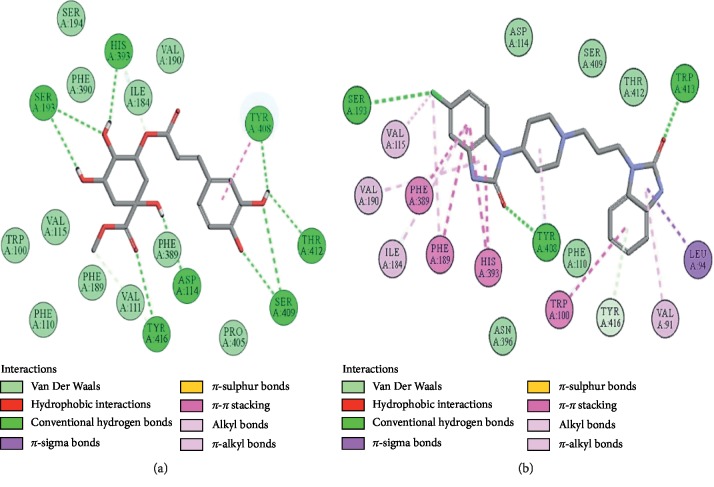
(a, b) The interactions of methyl chlorogenate and domperidone against the target dopaminergic D_2_ receptor, respectively, evaluated through Biovia Discovery Studio 2016.

**Figure 11:**
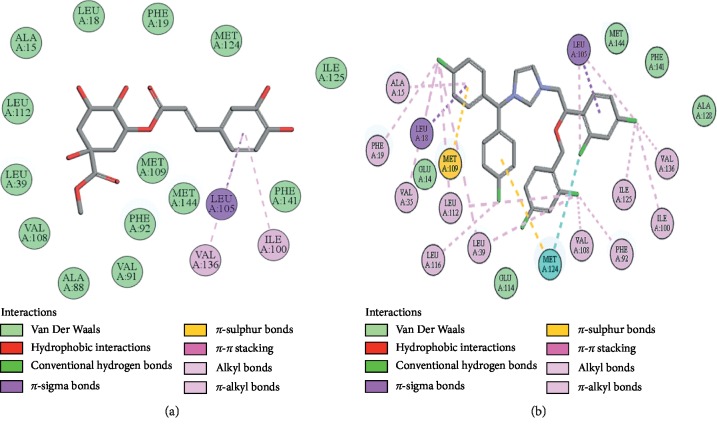
(a, b) The interactions of methyl chlorogenate and calmidazolium against the target calmodulin receptor, respectively, evaluated through Biovia Discovery Studio 2016.

**Figure 12:**
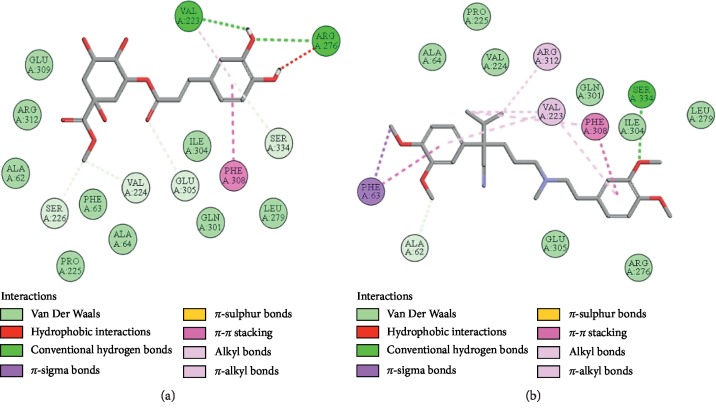
(a, b) The interactions of methyl chlorogenate and verapamil against the target voltage gated L-type calcium channels, respectively, evaluated through Biovia Discovery Studio 2016.

**Figure 13:**
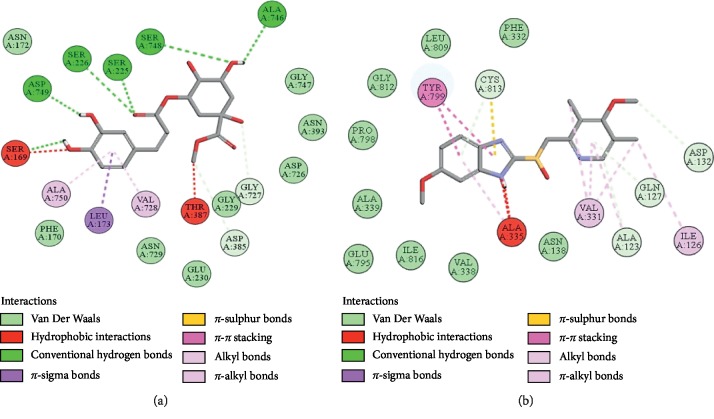
(a, b) The interactions of methyl chlorogenate and omeprazole against the target H^+^/K^+^ ATPase receptor, respectively, evaluated through Biovia Discovery Studio 2016.

**Figure 14:**
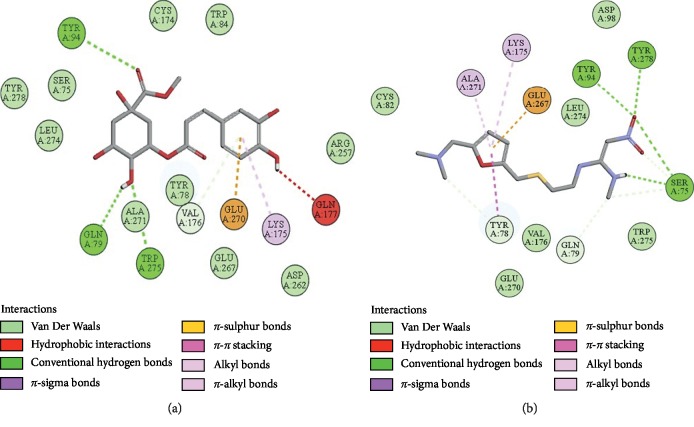
(a, b) The interactions of methyl chlorogenate and ranitidine against the target histaminergic H_2_ receptor, respectively, evaluated through Biovia Discovery Studio 2016.

**Figure 15:**
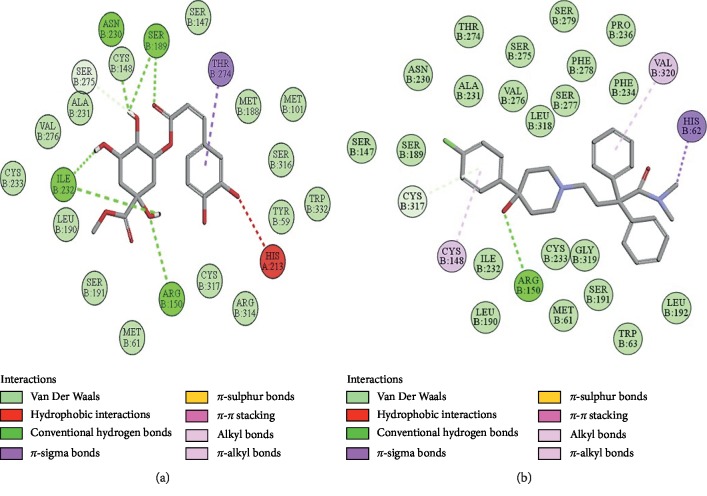
(a, b) The interactions of methyl chlorogenate and loperamide against the target mu-opioid receptor, respectively, evaluated through Biovia Discovery Studio 2016.

**Figure 16:**
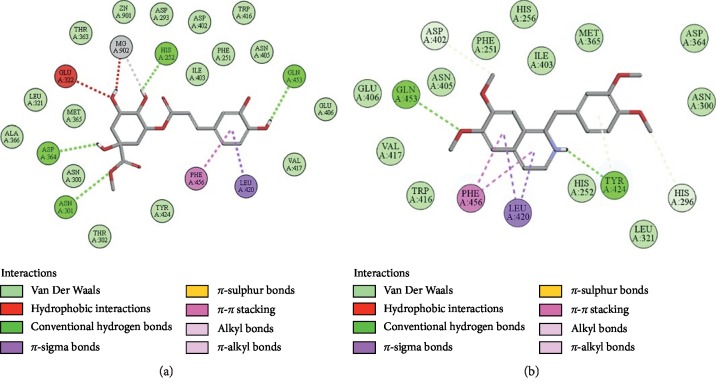
(a, b) The interactions of methyl chlorogenate and papaverine against the target phosphodiesterase enzyme, respectively, evaluated through Biovia Discovery Studio 2016.

Energy values and H-bonds are the two main contributing factors for docking evaluation. However, formation of *π*-*π* bonds, *π*-alkyl bonds, *π*-sigma bonds, and Vander Waal forces plays a leading role between ligand-receptor complexes. In this study, methyl chlorogenate showed variable binding affinities against different protein receptors.  Table 4 summarizes the atomic energy values (kcal/mol) and residues involved in H-bonding, *π*-*π* bonding, and other hydrophobic interactions of best dock poses of methyl chlorogenate and reference drugs in comparison with adrenergic *α*_1_ receptor, muscarinic M_1_, muscarinic M_3_, dopaminergic D_2_, calmodulin, mu-opioid, voltage gated L-type calcium channel, histaminergic H_2_, H^+^/K^+^ ATPase pump, and phosphodiesterase enzyme are plotted. Figures 7–16 illustrate the 2D view of interactions of methyl chlorogenate along with standard drugs. Against *α*_1_ adrenergic receptor, methyl chlorogenate showed *E*-value of −7.5 kcal/mol and formed 2 H-bonds and 4 hydrophobic interactions whereas phenylephrine showed *E*-value of −6.6 kcal/mol and formed 5 H-bonds, 1 *π*-*π* bonds, and 4 other hydrophobic interactions. The 2D interactions are shown in Figure 7. Against M_1_ muscarinic receptor, methyl chlorogenate showed *E*-value of −7.5 kcal/mol and formed 6 H-bonds, 2 *π*-*π* bonds, and 1 hydrophobic interaction, whereas pirenzepine showed *E*-value of −8.8 kcal/mol and formed 2 H-bonds, 1 *π*-*π* bond, and 1 other hydrophobic interaction. Its 2D interactions are shown in Figure 8. Against M_3_ muscarinic receptor, methyl chlorogenate showed *E*-value of −6.1 kcal/mol and formed 2 H-bonds and 5 hydrophobic interactions, whereas atropine showed *E*-value of −5.6 kcal/mol and formed 2 H-bonds, 1 *π*-*π* bond, and 19 other hydrophobic interactions as shown in 2D interactions in Figure 9. Against D_2_ dopaminergic receptor, methyl chlorogenate showed *E*-value of −7.8 kcal/mol and formed 9 H-bonds, 1 *π*-*π* bond, and 1 hydrophobic interactions, whereas domperidone showed *E*-value of −9.3 kcal/mol and formed 3 H-bonds, 5 *π*-*π* bonds, and 8 other hydrophobic interactions, as shown in Table 4, and 2D interactions as shown in Figure 10. Against calmodulin receptor, methyl chlorogenate showed *E*-value of −6.2 kcal/mol and formed 3 hydrophobic interactions, whereas calmidazolium showed *E*-value of −8.2 kcal/mol and formed 20 other hydrophobic interactions given in Table 4 and 2D interactions shown in Figure 11. Against calcium channel receptor, methyl chlorogenate showed *E*-value of −7.3 kcal/mol and formed 2 H-bonds, 1 *π*-*π* bond, and 6 hydrophobic interactions, whereas verapamil showed *E*-value of −6.7 kcal/mol and formed 1 H-bond, 2 *π*-*π* bonds, and 7 other hydrophobic interactions given in Table 4 and 2D interactions as shown in Figure 12. Against H^+^/K^+^ ATPase receptor, methyl chlorogenate showed *E*-value of −7.9 kcal/mol and formed 6 H-bonds and 7 hydrophobic interactions, whereas omeprazole showed *E*-value of −8.1 kcal/mol and formed 2 *π*-*π* bonds and 12 other hydrophobic interactions given in Table 4 and 2D interactions as shown in Figure 13. Against H_2_ histaminergic receptor, methyl chlorogenate showed *E*-value of −7.4 kcal/mol and formed 3 H-bonds and 4 hydrophobic interactions, whereas ranitidine showed *E*-value of −5.7 kcal/mol and formed 4 H-bonds, 1 *π*-*π* bond, and 7 other hydrophobic interactions given in Table 4 and 2D interactions as shown in Figure 14. Against mu-opioid receptor, methyl chlorogenate showed *E*-value of −9.2 kcal/mol and formed 6 H-bonds and 3 hydrophobic interactions, whereas loperamide showed *E*-value of −9.5 kcal/mol and formed 1 H-bond, and 4 other hydrophobic interactions given in Table 4 and 2D interactions as shown in Figure 15. Against phosphodiesterase enzyme receptor, methyl chlorogenate showed *E*-value of −8.9 kcal/mol and formed 4 H-bonds, 1 *π*-*π* bond, and 4 hydrophobic interactions, whereas papaverine showed *E*-value of −8.1 kcal/mol and 2 H-bonds, 2 *π*-*π* bonds, and 5 other hydrophobic interactions given in Table 4 and 2D interactions as shown in Figure 16.

## 4. Discussion

On the basis of ethnopharmacological utilization of *Manilkara zapota* in gastric disorders, such as gastritis, constipation, and diarrhea, *Manilkara zapota* extract was evaluated for its antidiarrheal, antisecretory, antispasmodic, charcoal meal gastrointestinal motility, and antiulcer effects. In vitro, in silico, and proteomic approach were used for the explication of possible underlying mechanisms to rationalize the aforementioned ethnomedicinal uses of the plant.

*Manilkara zapota* fruit extracts exhibited protective effects against castor oil-induced diarrhea, similar to the effect produced by loperamide, a standard drug, while its possible underlying mechanism was estimated through isolated tissue preparations also associated with the reduction in gastric motility. Castor oil is responsible for increasing intestinal fluid as well as diarrheal effect through its active metabolite, i.e., ricinoleic acid [10]. It changes the electrolyte and water transport and generates enormous contractions in the transverse and distal colon. Gastrointestinal motility is maintained through the release of various mediators which are histamine, prostaglandins, acetylcholine, substance P, 5-hydroxytrptamine, nitric oxide, and cholecystokinin which provides stimulatory effects in gut and ultimately increases cytosolic Ca^2+^ along with the release of mediators that block the above-described pathways accompanying with nonspecific inhibitory action that would relief gut disorders [11]. *Manilkara zapota* extracts demonstrated protective effect against castor oil-induced intestinal fluid secretion in mice. *Manilkara zapota* extracts contain a gut relaxant constituent which mediates antidiarrheal and antisecretory effects.

Papaverine (a standard phosphodiesterase enzyme (PDE) inhibitor), chloroform, and aqueous extracts of plant caused a dose-dependent inhibition of spontaneous and high K^+^-induced contractions in isolated rabbit preparations by producing a similar pattern of nonspecific inhibitory response whereas verapamil was found more potent against K^+^ (80 mM) induced contractions. These data proposed that plant extracts produce their relaxant effect through the dual mechanisms with CCB and producing relaxation effect, like PDE enzyme inhibition [18]. Antidiarrheal effect of the Mz fruit extract, mediated possibly through the dual blockade of phosphodiesterase enzyme receptors and Ca^2+^ channels, provides evidence for its effectiveness in diarrhea, though additional mechanisms can be ruled out. The phytochemical screening of *Manilkara zapota* fruit extract revealed the presence of different classes of chemicals, such as flavonoids, polyphenols, alkaloids, carbohydrates, saponins, and anthraquinones. Moreover, polyphenols target gut microbiota activity through its metabolites [19]; hence, the presence of these phytochemicals in *Manilkara zapota* may be accountable for its efficacy in diarrhea, though further research is required to explore the precise nature of chemical constituents mediating alleged biological activities. For charcoal meal transient time activity, *Manilkara zapota* extract in the small intestine produces suppression of the propulsion of charcoal marker in test doses just like standard drug atropine sulphate that has prominent anticholinergic effect on intestinal transit. A diminished GIT motility tone causes increase in the stay of substances in intestine, which permits better water absorption. These findings proposed that *Manilkara zapota* extract has an effect on the peristaltic movement of intestine which indicates its antimotility effects. In gastrointestinal tract, various aggressive and protective factors play an important role in the production and release of acids. Disturbance in these factors results in rupturing of mucosal protection which exposes gastric lining to different enzyme and acid productions leading to the sores called ulcers [20]. Ethanol-induced gastric model was used to explore the beneficial effect of *Manilkara zapota* extract through a variety of mechanisms that stimulate ulcers including free radicals OH^−^, NO production, mucus exhaustion, mucosal damage, release of superoxide anion, which ultimately prolonged the tissue oxidative stress, and release of inflammatory mediators like TNF-*α*, p-NF*κ*B, and COX-2, which are measured through the proteomic analysis. The potential of *Manilkara zapota* extract to produce antiulcer effect might be due to its CCB effect, as Ca^2+^ antagonist is well known to demonstrate such effects [2]. In pathophysiology of gastric ulcers, oxidative stress plays a vital role. Antioxidant and nitric oxide free radical scavenging activity has been reported by *Manilkara zapota*, which may be responsible for its effectiveness as antiulcer agent [3]. Furthermore, ELISA was done to quantify the protein expression of IL-18, and Mz.Aq significantly attenuates necrosis in ulcerative tissue as compared to Mz.CHCl_3_ achieved through proteomic analysis [15]. Mz.Aq extract administration exerts more protective effect as compared to Mz.CHCl_3_ in ulcerative model, so, for IHC and H&E staining, Mz.Aq was selected for further analysis. Mz.Aq protects the gastric cells through the inhibition of inflammatory markers like TNF-*α*, p-NF*κ*B, and COX-2. The results were quite significant as compared to ethanol. TNF-*α*, COX-2, and p-NF*κ*B are widely considered as prototypic proinflammatory cytokines due to their principal role in initiating the cascade of the activation of other cytokines and growth factors in the inflammatory response [20]. After injury or during inflammation, TNF-*α* and NF*κ*B signaling pathway is activated which plays a vital role in inflammation. Ethanol induces inflammation in gastric mucosa in the control group, whereas *Manilkara zapota* extracts decreased the levels of TNF-*α*, p-NF*κ*B, and COX-2 as compared to omeprazole when given alone to rats. *Manilkara zapota* extracts have broader therapeutic index as no mortality was observed at a dose of 5 g/kg.

The ligand methyl chlorogenate (phytochemical constituent) was docked in the catalytic active pocket of *α*_1_-adrenergic receptor, muscarinic M_1_ and M_3_, domperidone D_2_, calmodulin, voltage gated L-type calcium channel, gastric hydrogen potassium ATPase, histamine H_2_, mu-opioid, and phosphodiesterase enzyme receptor targets that may be possibly associated with the pathophysiology of gastric disorders [21]. Docking is used as a preliminary tool used to evaluate the affinity of ligands to their particular protein targets. Molecular docking has a significant role in drug discovery and development, which includes structure-based evaluation and finding target specificity along with binding affinity. For virtual screening, DSV was used through PyRx [22]. It has gradient optimization method and it predicts the binding mode of ligand-receptor complex more precisely. Affinity of ligands with receptor could be assessed through *E*-value and hydrogen bonding that relates their influential effect in gastrointestinal diseases [23]. Based on *E*-value against different protein targets, the order of ligand affinity was found as mu-opioid > phosphodiesterase enzyme > H^+^/K^+^ATPase > D_2_ > *α*_1_ > M_1_ > H_2_ > voltage gated L-type calcium channel > Calmodulin receptor > M_3_.

## 5. Conclusions

*Manilkara zapota* Linn. holds a vital place in the traditional system of medicines. The current explored potential of the plant signifies its importance to the pharmaceutical industry due to exhibited antidiarrheal, antisecretory, antispasmodic, antimotility, and antiulcer effects. Most histopathological changes in gastric tissues and associated derangement in related polyclonal antibodies were largely prevented by the *Manilkara zapota* extract, thus paving the way to a new therapeutic approach towards the management of inflammation‐induced gastric mucositis. The plant constituent methyl chlorogenate showed moderate binding affinities (*E*-value ≥ −6.1 kcal/mol) against muscarinic M_1_, muscarinic M_3_, histaminergic H_2_ receptors, H^+^/K^+^ ATPase pump, calmodulin, adrenergic *α*_1_, and dopaminergic D_2_ receptors and voltage gated L-type calcium channels, while it showed high affinities (*E*-value ≥ 8 kcal/mol) against mu-opioid and phosphodiesterase enzyme.

## Figures and Tables

**Table 1:** Effect of *Manilkara zapota* extracts: chloroform (Mz.CHCl_3_), aqueous (Mz.Aq), and loperamide against castor oil-induced diarrhea (10 mL/kg) in mice.

Treatment	No. of mice (out of 5) with diarrhea	Protection (%)
Saline (10 mL/kg) + castor oil	5/5	0
Mz.CHCl_3_ (50 mg/kg) + castor oil	2/5^*∗*^	60
Mz.CHCl_3_ (100 mg/kg) + castor oil	1/5^*∗*^	80
Mz.CHCl_3_ (300 mg/kg) + castor oil	0/5^*∗∗*^	100
Mz. Aq (50 mg/kg) + castor oil	4/5	20
Mz.Aq (100 mg/kg) + castor oil	3/5	40
Mz. Aq (300 mg/kg) + castor oil	2/5^*∗*^	60
Loperamide (10 mg/kg) + castor oil	0/5^*∗∗*^	100

^*∗*^ *p* < 0.05 and ^*∗∗*^*p* < 0.01 versus saline + castor oil-treated group (*Χ*^2^-test).

**Table 2:** Effect of *Manilkara zapota* extracts: chloroform (Mz.CHCl_3_), aqueous (Mz.Aq), and atropine on charcoal meal transit time in rats.

Doses (mg/kg, p.o.)	Mean length of intestine (cm)	Distance moved by charcoal (cm)	% intestinal transient	% inhibition
Saline (10 mL/kg)	92.6 ± 1.6	90 ± 1.3	97.1	—
Mz.CHCl_3_ (50 mg/kg)	87.4 ± 1.3	76.6 ± 1.9^*∗∗∗*^	87.64	9.74
Mz.CHCl_3_ (100 mg/kg)	90.8 ± 0.8	70.6 ± 0.8^*∗*^	77.75	19.92
Mz.CHCl_3_ (300 mg/kg)	84.4 ± 0.5	59.8 ± 0.3^*∗∗∗*^	70.85	27.03
Mz.Aq (50 mg/kg)	73.6 ± 0.9	86.6 ± 0.2	84.98	12.48
Mz.Aq (100 mg/kg)	74.2 ± 0.5	57 ± 0.7^*∗*^	76.81	20.89
Mz.Aq (300 mg/kg)	77 ± 0.7	55.4 ± 0.9^*∗∗*^	71.94	25.91
Atropine (0.1 mg/kg, i.p.)	90.8 ± 0.9	16.4 ± 0.6^*∗∗∗*^	18.06	81.40

^*∗*^ *p* < 0.05, ^*∗∗*^*p* < 0.01, and ^*∗∗∗*^*p* < 0.001 compared to control saline group. One-way analysis of variance followed by Tukey's post hoc test, *n* = 5.

**Table 3:** Protective effect of *Manilkara zapota* extracts: chloroform (Mz.CHCl_3_), aqueous (Mz.Aq), and omeprazole against ethanol (1 mL/100 g) induced gastric ulcers in rats.

Treatment	Ulcer index	% inhibition
Saline 10 mL/kg + ethanol	10.0 ± 0.0	—
Mz.CHCl_3_ (50 mg/kg) + ethanol	8.0 ± 0.31^*∗*^	20
Mz.CHCl_3_ (100 mg/kg) + ethanol	4.2 ± 0.2^*∗∗*^	58
Mz.CHCl_3_ (300 mg/kg) + ethanol	2.4 ± 0.44^*∗∗∗*^	76
Mz.Aq (50 mg/kg) + ethanol	6.2 ± 0.3^*∗∗∗*^	38
Mz.Aq (100 mg/kg) + ethanol	4.4 ± 0.14^*∗∗∗*^	56
Mz.Aq (300 mg/kg) + ethanol	2.4 ± 0.24^*∗∗∗*^	76
Omeprazole (20 mg/kg) + ethanol	1.4 ± 0.24^*∗∗∗*^	86

^*∗*^ *p* < 0.05, ^*∗∗*^*p* < 0.01, and ^*∗∗∗*^*p* < 0.001 compared to control saline group. One-way analysis of variance, followed by Tukey's post hoc test, *n* = 5.

**Table 4:** Binding energy (kcal/mol) and post dock analysis of the best conformational pose of methyl chlorogenate with adrenergic *α*_1_ receptor, muscarinic M_1_, muscarinic M_3_, dopaminergic D_2_, calmodulin, mu-opioid, voltage gated L-type calcium channel, H^+^/K^+^ ATPase pump, histaminergic H_2_, and phosphodiesterase enzyme.

Target proteins	Methyl chlorogenate						Standard drugs						
	*E*-value (kcal/mol)	H-bonds	Amino acids forming H-bonds	*π*-*π* bonds	Amino acids forming *π*-*π* bonds	Amino acids forming other hydrophobic interactions	Standard drugs	*E*-value (kcal/mol)	H-bonds	Amino acids forming H-bonds	*π*-*π* bonds	Amino acids forming *π*-*π* bonds	Amino acids forming other hydrophobic interactions
Adrenergic *α*_1_	−7.5	2	SER 158 GLN 177	0	—	LEU 162ALA 103CYS 99PRO 161	Phenylephrine	−6.6	5	THR 111 SER 192 CYS 110 ASP 106 TYR 316	1	PHE 298	VAL 107(2) CYS 110 PHE 312
Muscarinic M_1_	−7.5	6	TYR 82(2) TYR 106(2) TYR 381 ILE 180	2	TYR 404(2)	THR 189	Pirenzepine	−8.8	2	THR 189 TYR 381	1	TRP 400	LEU 183
Muscarinic M_3_	−6.1	2	GLU 6 ASN 9	0	—	GLY 15 GLU 4 GLU 8 THR 7 ASN 9	Atropine	−5.6	2	GLU 6 THR 7	1	PHE 10	ALA 5 GLU 8(11) PRO 13(2) ASN 9(2) THR 7(3)
Dopaminergic D_2_	−7.8	9	ASP 114 TYR 416 SER 409(2) THR 412 TYR 408 SER 193(2) HIS 393	1	TYR 408	ILE 184	Domperidone	−9.3	3	SER 193 TYR 408 TRP 413	5	PHE 389 PHE 189 HIS 393(2) TRP 100	VAL 115 VAL 190 ILE 184 PHE 189 TYR 408 TYR 416 VAL 91 LEU 94
Calmodulin	−6.2	0	—	0	—	ILE 100 VAL 136 LEU 105	Calmidazolium	−8.2	0	—	0	—	LEU 105(3) VAL 136 ILE 100 ILE 125 PHE 92 VAL 108 MET 124(2) LEU 39(2) LEU 116 LEU 112(2) MET 109 LEU 18 VAL 35 PHE 19 ALA 15
Calcium channel	−7.3	2	VAL 223 ARG 276	1	PHE 308	SER 226 VAL 224 GLU 305 SER 334 VAL 223 ARG 276	Verapamil	−6.7	1	SER 334	2	PHE 308 PHE 63	PHE 63 ARG 312 VAL 223(3) PHE 308 ALA 62
H^+^/K^+^ ATPase	−7.9	6	ASP 749 SER 226 SER 225 SER 748 ALA 746 SER 169	0	—	GLY 727 ASP 385 THR 387 VAL 728 LEU 173 ALA 750 SER 169	Omeprazole	−8.1	0	—	2	TYR 799(2)	CYR 213(2) ALA 335(2) VAL 331(3) ALA 123(2) GLN 127 ILE 126 ASP 132
Histaminergic H_2_	−7.4	3	GLN 79 TRP 275 TYR 94	0	—	VAL 176 GLU 270 LYS 175 GLN 177	Ranitidine	−5.7	4	SER 75(2) TYR 94 TYR 278	1	TYR 78	ALA 271 LYS 175 GLU 267 SER 75(2) GLN 79 TYR 78
Mu-opioid	−9.2	6	ILE 232(2) ARG 150 SER 189(2) CYS 148	0	—	SER 275 THR 274 HIS 213	Loperamide	−9.5	1	ARG 150	0	—	VAL 320 HIS 62 CYS 317 CYS 148
Phosphodiesterase enzyme	−8.9	4	ASP 364 ASN 301 HIS 252 GLN 453	1	PHE 456	GLU 322MG 902(2) LEU 420	Papaverine	−8.1	2	GLN 453 TYR 424	2	PHE 456(2)	ASP 402 LEU 420(2) TYR 424 HIS 296

ALA, alanine; ASN, asparagine; ASP, aspartic acid; ARG, arginine; CYS, cysteine; GLU, glutamic acid; GLY, glycine; GLN, glutamine; HIS, histidine; LYS, lysine; PHE, phenylalanine; SER, serine; THR, threonine; TRP, tryptophan; TYR, tyrosine; VAL, valine.

## Data Availability

The data used to support the findings of this study are available from the authors upon request.
